# Metabolism of plasma cholesterol and lipoprotein parameters are related to a higher degree of insulin sensitivity in high HDL-C healthy normal weight subjects

**DOI:** 10.1186/1475-2840-12-173

**Published:** 2013-11-22

**Authors:** Camila C Leança, Valéria S Nunes, Natália B Panzoldo, Vanessa S Zago, Eliane S Parra, Patrícia M Cazita, Matti Jauhiainen, Marisa Passarelli, Edna R Nakandakare, Eliana C de Faria, Eder CR Quintão

**Affiliations:** 1 Lipids Laboratory (LIM-10), Endocrinology and Metabolism Division of Hospital das Clinicas, Faculty of Medical Sciences, University of Sao Paulo, Av. Dr. Arnaldo, 455 - room 3305, Sao Paulo CEP 01246-00, Brazil; 2 Department of Clinical Pathology, School of Medical Sciences, State University of Campinas-UNICAMP, P.O. Box 6111, CEP 13083-970, Campinas, SP, Brazil; 3 National Institute for Health and Welfare, Public Health Genomics Research Unit, Helsinki, Finland

**Keywords:** Insulin resistance, HDL-C concentration, Lipoprotein lipases, Lecithin cholesterol acyl transferase, Pre-beta1 HDL, Plasma cholesterol metabolism markers, Cholesteryl ester transfer protein, Phospholipid transfer protein

## Abstract

**Background:**

We have searched if plasma high density lipoprotein-cholesterol (HDL-C) concentration interferes simultaneously with whole-body cholesterol metabolism and insulin sensitivity in normal weight healthy adult subjects.

**Methods:**

We have measured the activities of several plasma components that are critically influenced by insulin and that control lipoprotein metabolism in subjects with low and high HDL-C concentrations. These parameters included cholesteryl ester transfer protein (CETP), phospholipid transfer protein (PLTP), lecithin cholesterol acyl transferase (LCAT), post-heparin lipoprotein lipase (LPL), hepatic lipase (HL), pre-beta-_1_HDL, and plasma sterol markers of cholesterol synthesis and intestinal absorption.

**Results:**

In the high-HDL-C group, we found lower plasma concentrations of triglycerides, alanine aminotransferase, insulin, HOMA-IR index, activities of LCAT and HL compared with the low HDL-C group; additionally, we found higher activity of LPL and pre-beta-_1_HDL concentration in the high-HDL-C group. There were no differences in the plasma CETP and PLTP activities.

**Conclusions:**

These findings indicate that in healthy hyperalphalipoproteinemia subjects, several parameters that control the metabolism of plasma cholesterol and lipoproteins are related to a higher degree of insulin sensitivity.

## Background

The low plasma concentration of high density lipoprotein cholesterol (HDL-C) is an independent risk factor for ischemic heart disease and has complex multifactorial causes involving the actions of many genes. HDL particles have several functions, including reverse cholesterol transport and antioxidant, anti-inflammatory and antithrombogenic activities. The metabolic disturbances that cause variations in the plasma HDL-C concentration have been investigated in the genetic and secondary causes of dyslipidemia, such as obesity, insulin resistance, metabolic syndrome, ischemic heart disease and under the effect of several pharmacological agents [1]. Nevertheless, the precise mechanisms by which HDL particles prevent atherosclerosis are unknown. There has been considerable effort in utilizing genome-wide association to conclude that a genetic variant near IRS1 is associated with type 2 diabetes, insulin resistance and hyperinsulinemia. However, no clues have ever been provided with regard to the primary cause of the above mentioned associations with the regulation of the plasma HDL concentration or metabolism rate, leaving aside rates of cholesterol metabolism [2].

The plasma HDL-C concentration may play a role as an independent contributor to the pathogenesis of type 2 diabetes mellitus [3]. Premature atherosclerosis is related to several HDL-dependent activities that include a diminished cell cholesterol efflux capacity by HDL [4]. Interestingly, the ABCA1 receptor is considered a major factor in the maintenance of the plasma HDL-C concentration and for cell cholesterol efflux essential to prevent pancreatic islet lipid accumulation [5] and carriers of loss-of-function mutations in ABCA1 show impaired insulin secretion with [6] or without insulin resistance [7]. Also, certain ABCA1 genotypes have been related to increased risk of diabetes mellitus in humans with [8,9] or without [10] low HDL concentrations in plasma. Mice lacking *Abca1* have impaired glucose tolerance [11] although in the LDLr knockout mouse model no associations were observed between the quantitative trait loci of atherosclerosis and levels of total cholesterol, HDL-C, insulin or body weight [12]. Also, in non-insulin dependent diabetes mellitus pancreatic beta cells may fail to compensate for insulin resistance and the ABCA1 receptor, a cellular cholesterol transporter, plays a role as a regulator of cholesterol homeostasis and insulin secretion in these cells [13]. Furthermore, HDL exerts insulin-sensitizing effects enhancing the glucose disposal in type 2 diabetes mellitus patients [14].

Several investigations have dealt with the interactions of dyslipidemia, insulin resistance and HDL lipidome. However, these investigations have invariably been biased by other factors that independently interfere with the metabolism of HDL, such as variations in the body mass index, waist circumference, plasma triglyceride concentration, and age or gender differences between the investigated groups [15-18]. These variables make it impossible to ascertain a cause/effect relationship between the HDL concentration and insulin sensitivity.

High cholesterol synthesis and low cholesterol absorption have been associated with metabolic syndrome [19], insulin resistance [20] and type 2 diabetes, independently of body weight [21]. We have previously shown that healthy, non-obese adults with a high plasma HDL-C concentration have greater plasma concentrations of a cholesterol synthesis marker (lathosterol) and diminished concentrations of intestinal dietary cholesterol absorption markers (campesterol and β-sitosterol) compared with low-HDL-C participants [22].

According to recent publications the relationship of plasma HDL-C and insulin activity seems more complex than previously reported. HDL-C plasma concentration was related to angiopoietin-3 like (Angptl3), which is an inhibitor of lipoprotein lipase activity, and to insulin sensitivity [23]. Also, HDL-C concentration may be influenced by insulin as suggested by a study in China relating lipoprotein lipase, vitamin D and insulin resistance [24]. In fact, insulin sensitivity likely explains the role of insulin on plasma HDL-C concentration because patients with insulinoma without insulin resistance that had been matched with controls for age and BMI do not increase the production rate of high density lipoprotein apolipoprotein AI which seems to be more dependent on the apoAI fractional catabolic rate [25].

We have then hypothesized that plasma HDL-C concentrations independently of an increased body mass index, age or gender might influence insulin sensitivity. Searching for possible causes we measured the activities of cholesteryl ester transfer protein (CETP), phospholipid transfer protein (PLTP), lecithin:cholesterol acyltransferase (LCAT), post-heparin lipoprotein lipase (LPL) and hepatic lipase (HL) in normal weight healthy adult subjects with low and high plasma HDL-C concentrations.

## Methods

### Subjects

Healthy volunteers (n = 73, 41 men and 32 women) with a plasma HDL-C concentration < 40 mg/dL or > 60 mg/dL, between the ages of 20 – 74 years, and with a BMI < 30 Kg/m^2^ were selected from the following institutions: the Lipid Clinic Disorders Service of the Endocrinology and Metabolism Unit of the Clinical Hospital of the Faculty of Medicine, University of São Paulo (HCFMUSP); the Clinical Hospital at the University of Campinas (UNICAMP); health centers of the city of Campinas; and the Laboratory of Ouro Verde Hospital, Campinas, São Paulo State, Brazil. All participants were informed of the objectives of the study and signed an informed written consent in accordance with the research protocols approved by the Ethics Committee of HCFMUSP and UNICAMP. The cases included in this study had previously been reported [22]. The exclusion criteria were obesity, metabolic syndrome, diabetes mellitus, uncontrolled thyroid function disorder, liver and kidney failure, smoking, alcohol abuse and the use of medications that may interfere with cholesterol metabolism, such as hormonal replacement therapy and blood lipid-lowering drugs.

### Measurements

Blood was drawn after a 12 h-fasting period. The blood samples were treated with 10% EDTA, and the plasma was immediately separated for further analysis. Plasma glucose, cholesterol and triglyceride concentrations were measured using commercially available enzymatic-colorimetric methods (Labtest Diagnostica, Brazil and Roche Diagnostics Corp, Indianapolis, IN, respectively). The plasma LDL-C concentration was calculated using the Friedewald formula [26].

The plasma insulin concentration was measured by an immunofluorometric assay using AutoDELFIA (Perkin Elmer Inc., Waltham, Mass.,USA), and the homeostasis model assessment insulin resistance (HOMA-IR) was calculated according to the following formula: Glucose (mmol/L) × Insulin (mU/L)/22.5. The alanine aminotransferase (ALT) and aspartate aminotransferase (AST) activities were measured by the kinetic method, and the high sensible plasma C-reactive protein (hsCPR) concentration was measured by immunoturbidimetric assay. The serum amyloid A (SAA) concentration was measured by nephelometry (Siemens Healthcare Diagnostics, Deerfield, IL, USA), and the levels of apolipoproteins were measured by immunoturbidimetric assays (Randox, Crumlin Co., Antrin, Ireland). The plasma concentrations of markers of intestinal cholesterol absorption (campesterol and β-sitosterol) and of cholesterol synthesis (desmosterol and lathosterol) that had been presented in our previous publication [22] were measured by gas chromatography (GC) coupled to a mass spectrophotometer (MS) (Shimadzu GCMS-QP2010 Plus, Kyoto, Japan), with the software GCMS solution ver. 2.5 [27] and are included in the present report. The LCAT-mediated cholesterol esterification rate was measured using endogenous substrates labeled with ^14^C-cholesterol, according to Dobiasova *et al.*[28], and the CETP activity which indirectly indicates CETP mass was measured by a radioisotopic assay using an exogenous substrate, as previously described [22]. The PLTP activity was measured using an exogenous radiometric assay, as described by Jauhiainen and Ehnholm [29]. HL and LPL activities were measured using a method adapted from Ehnholm [30]. Briefly, the plasma samples were drawn 15 minutes after intravenous heparin administration (100 U/Kg of body weight), and the lipase activities were measured via liberated free fatty acids using radiolabeled triolein emulsion as the substrate and 1 M NaCl as the LPL inhibitor. The pre-beta_1_-HDL concentration was measured using an enzyme-linked immunosorbent assay (ELISA) from Daiichi Pure Chemicals Co. (Tokyo, Japan).

### Statistical analysis

The statistical analyses were performed using GraphPad Prism version 4.0 (GraphPad Softwares, USA). Comparisons between groups were performed using Student *t* test. The significance level chosen was p < 0.05, and the results are presented as mean ± SD. A correlation matrix study was performed with GraphPad Instat version 3.05 (GraphPad Softwares, USA).

## Results

Anthropometric and laboratorial data are presented in Table 1. The plasma concentration of triglycerides (TG), insulin, HOMA-IR index and ALT were lower in the high-HDL group compared with the low-HDL-C group. As expected, the apolipoprotein (apo) AI and apo E concentrations were higher in the high-HDL-C group. For being slightly older than the hypoalphalipoproteinaemics a diminished degree of insulin sensitivity should have been expected in our hyperalphalipoproteinaemics and yet the opposite was found which reinforces the conclusion that our results were not biased by age differences between the groups.

**Table 1:** **Anthropometric data, blood chemistry and lipoprotein metabolism data in healthy participants with low plasma HDL-C *vs. *** **high plasma HDL-C concentration**

	**Low HDL**	**High HDL**	***P***
Gender	21 men/16 women	20 men/16 women	
Age (year)	37 ± 11	45 ± 14	0.022
BMI (Kg/m^2^)	24.0 ± 2.4	23.5 ± 3.3	
Waist (cm)	80 ± 9	80 ± 9	
Glucose (mg/dL)	83 ± 8	84 ± 8	
Insulin (μU/mL)	6.5 ± 3.1	4.4 ± 1.8	<0.001
HOMA index	1.35 ± 0.67	0.91 ± 0.39	0.001
Triglycerides (mg/dL)	97 ± 33	71 ± 34	0.002
Cholesterol (mg/dL)	159 ± 25	186 ± 30	<0.001
HDL-C (mg/dL)	34 ± 4	79 ± 13	<0.001
LDL-C (mg/dL)	106 ± 22	93 ± 22	0.018
VLDL (mg/dL)	19 ± 7	14 ± 7	0.001
TG/HDL ratio	3.00 ± 1.32	0.91 ± 0.42	<0.001
Apo AI (mg/dL)	84.8 ± 15.5	124.9 ± 19.4	<0.001
Apo B (mg/dL)	75.4 ± 21.7	71.8 ± 17.1	
Apo CII (mg/dL)	3.1 ± 1.9	3.1 ± 1.5	
Apo CIII (mg/dL)	7.3 ± 3.1	6.7 ± 4.4	
Apo E (mg/dL)	2.1 ± 0.8	3.2 ± 1.2	<0.001
ALT (U/L)	24 ± 16	16 ± 5	0.005
AST (U/L)	20 ± 6	19 ± 3	
hsCRP (mg/L)	1.7 ± 2.0	2.1 ± 4.6	
SAA (mg/L)	3.2 ± 2.8	6.4 ± 10.5	
CETP (%CE)	13.6 ± 5.3	13.5 ± 4.5	
PLTP (nmolPC/mL/h**)**	6,247 ± 1,851	6,439 ± 1,617	
LCAT (%CE)	3.71 (2.48-5.02)	2.68 (1.63-3.61)	0.011
LPL (nmFFA/mL/h)	2,403 ± 1,304	4,597 ± 2,616	<0.001
HL (nmFFA/mL/h)	4,517 ± 1,622	3,159 ± 1,424	<0.001
Pre-beta_1_-HDL (ng/mL)	11.8 ± 5.7 (n = 33)	22.4 ± 22.5 (n = 34)	0.008

*ALT*, Alanine aminotransferase. *Apo*, Apolipoprotein. *AST*, Aspartate aminotransferase. *BMI*, Body mass index. *CETP*, Cholesteryl ester transfer protein. *HL*. Hepatic lipase. *HOMA*, Homeostasis model assessment. *LCAT*, Lecithin: cholesterol acyltransferase. *LPL*, Lipoprotein lipase. *hsCPR*, C- reactive protein. *SAA*, Serum amyloid A. *PLTP*, Phospholipid transfer protein. Student *t* test, low HDL-C *vs*. high HDL-C. Data are presented as mean ± SD. *P* < 0.05. Pre-beta_1_-HDL (n = 33 low HDL-C; n = 34 high HDL-C). PLTP, HL and LPL (n = 34 low HDL-C; n = 66 high HDL-C).

The high-HDL-C group presented lower LCAT and post-heparin hepatic lipase activities and higher post-heparin lipoprotein lipase activity and pre-beta-_1_-HDL concentration than did the low-HDL-C group. Plasma LCAT activity significantly reduced in the hyperalphalipoproteinaemic cases compared with the hypoalphalipoproteinaemic cases is congruent with the elevated plasma LCAT activity found in metabolic syndrome attributed to insulin resistance [31]. These results are supported by a report of LCAT-null mice being protected from both diet-induced obesity and insulin resistance and from simultaneously developing increased hepatic insulin sensitivity [32]. The plasma exogenous CETP and PLTP activities did not vary between our two HDL-C groups and therefore did not explain their different HDL-C concentrations. The latter is likely due to the combination of the remarkable differences in the post-heparin lipoprotein and hepatic lipase activities in addition to a modest, although significant, LCAT variation.

In an attempt to elucidate the origin of the metabolic processes involved, a univariate correlations of all the parameters measured was performed. Table 2 presents only the results that were highly significant (greater than ± 0.5, p < 0.05), and therefore capable of indicating meaningful dependent relationships. Certain significant relationships were predictable according to the literature, such as the waist circumference and body mass index (BMI) (a), the effect of age raising the plasma LDL-C concentration (b), lowering the post-heparin lipoprotein lipase (c) [33], an inverse association of plasma TG and HDL-C concentrations (d) as well as a marker of insulin resistance (HOMA-IR) varying directly with the waist circumference (e) [34] and inversely with post-heparin LPL activity (f) [35]. LPL varied inversely with the average arterial blood pressure (g), reinforcing the state of insulin resistance typical of the metabolic syndrome that characterized the low-HDL-C cases; however, potential genetic reasons for this association cannot be ignored [36]. ALT activity associated with the marker of cholesterol body synthesis (desmosterol) (h) likely reinforces the enhanced insulin sensitivity [37] in the high-HDL-C cases. However, as a marker of cholesterol synthesis, desmosterol alone did not differ between the HDL-C concentration groups in our previous investigation, which served as a database for the present report [22]. In the latter study, plasma campesterol (i) was shown to be one of the two markers of cholesterol absorption. Its concentration was greater in the high-HDL-C group than in the low-HDL-C group. The plasma campesterol concentration is shown here to correlate with the participants’ age, characterizing a new, interesting finding because the absorption data had been properly corrected for the plasma cholesterol concentration and body mass index. In contrast, lathosterol, which is a significant marker of whole-body cholesterol synthesis, was not only elevated in the low-HDL-C cases but also correlated with the plasma LDL-C concentration (j).

**Table 2:** **Linear regression analyses of all significant correlations (*P *** **<0.05)**

**Variable**	***r***
a) BMI × waist	0.5669
b) LDL-C × age	0.5098
c) LPL × age	−0.5052
d) Triglycerides × HDL-C	−0.6227
e) HOMA-IR × waist	0.5700
f) HOMA-IR × LPL	−0.5270
g) LPL × Blood Pressure (average)	−0.5154
h) Desmosterol × ALT	0.5642
i) Campesterol × age	0.5141
j) Lathosterol × LDL-C	0.581

*ALT*, Alanine aminotransferase. *BMI*, Body mass index. *HOMA*, Homeostasis model assessment. *LPL*, Lipoprotein lipase.

## Discussion

As recently reviewed, HDL-C is controlled simultaneously by several factors, such as LCAT, PLTP, CETP, and peripheral and hepatic post-heparin lipoprotein lipases, which by themselves are regulated by insulin [38], as shown in the present study, in addition to the angiopoietin-3-like gene as indicated in a recent study [23].

Challenging the notion that LCAT is needed for an efficient atheroprotection, no association between carotid intima/media thickness (IMT) and LCAT was observed in men, and carotid IMT increased with LCAT quartiles in women [39]. However, in a Danish population, ischemic heart disease was associated with low LCAT and high pre-beta_1_-HDL concentrations [40]. In contrast, our hyperalphalipoproteinaemia cases showed low LCAT activity and high pre-beta_1_-HDL. This result was expected given the diminished LCAT conversion of the precursor pre-beta_1_-HDL into the larger HDL likely being overcome by a fast generation of pre-beta_1_-HDL and HDL secondarily to an increased post-heparin lipoprotein lipase activity. Our observation that the hyperalphalipoproteinaemic participants have increased post-heparin lipoprotein lipase activity and diminished hepatic lipase activity compared with the hypoalphalipoproteinaemic participants is in agreement with a previous report [41]. Due to the high-HDL insulin-sensitive state in hyperalphalipoproteinaemia binding and internalization rates of triglyceride-enriched HDL may slow down [42]. Thus, our results conform to the following chain of events described in a previous review [43]. In insulin-sensitive cases - as our high-HDL-C participants - the consequent enhanced post-heparin lipoprotein lipase activity induces HDL production that, combined with the diminished hepatic lipoprotein lipase, further increases the HDL particle size and quantity. In hyperalphalipoproteinemia cases, we found a diminished TG/HDL-C plasma ratio that has been deemed as critically dependent on insulin activity [44]. Although the plasma CETP activity measured by the exogenous method that reflects the plasma CETP concentration [22] did not differ between the two groups the low TG/HDL-C likely is consequent to decreased endogenous CETP activity due to a diminished triglyceride availability from apoB-containing LP for exchange with HDL cholesteryl ester in hyperalphalipoprotaeinemia cases.

In agreement with a primary role for insulin sensitivity in cholesterol metabolism and, to a considerable extent, in plasma HDL-C concentration variation, we found a diminished ALT level within the “reference” range in hyperalphalipoprotaeinemia cases, a result compatible with other reports relating ALT to insulin sensitivity [37].

Even in the absence of diabetes mellitus, changes in cholesterol metabolism are important predictors of cardiovascular disease, as has been recently shown Weingärtner *et al.*[45], however, that work did not investigate the relationship with insulin resistance or with plasma HDL-C concentrations. Furthermore, there has been clinical evidence in healthy, lean adult humans supporting insulin sensitivity-dependency on the abdominal fat quantity simultaneously with plasma HDL-C concentration variations [46]. Our present and previous findings [22] indicate that, in hyperalphalipoproteinemia, variations in several parameters that control the plasma lipoprotein metabolism are related to an increased degree of insulin sensitivity. In support of this conclusion, a study in India on normal-weight offspring with type 2 diabetes mellitus that were matched with controls of comparable age showed that the former had lower plasma HDL-C concentrations and higher values of plasma insulin [47]. The latter finding suggests that the development of diabetes mellitus is preceded by such changes, although not linked to any specific genetic cause.

Further investigation is required utilizing more reliable methods to measure insulin sensitivity in these case, such as the clamp technique.

## Abbreviations

(LPL): HDL-C, Lipoprotein lipase; (LCAT): Lecithin cholesterol acyl transferase; HDL: Pre-beta1; (CETP): Cholesteryl ester transfer protein; (PLTP): Phospholipid transfer protein; (IRS1): Insulin receptor; (ALT): Alanine amino-transaminase; (IMT): Intima/media thickness; (AST): Aspartate aminotransferase; (BMI): Body mass index; (HL): Hepatic lipase; (HOMA): Homeostasis model assessment; (hsCPR): C- reactive protein; (SAA): Serum amyloid A.

## Competing interests

The authors declare that they have no competing interest.

## Authors’ contributions

CCL researched data and wrote manuscript, VSN researched data, NBP researched data, VSZ researched data, ESP researched data, PMC researched data, MJ researched data, MP contributed to discussion and reviewed/edited manuscript, ERN contributed to discussion and reviewed/edited manuscript, ECF contributed to discussion and reviewed/edited manuscript, ECRQ contributed to discussion and wrote manuscript. All authors read and approved the final manuscript.
